# Microwave ablation with local pleural anesthesia for subpleural pulmonary nodules: our experience

**DOI:** 10.3389/fonc.2022.957138

**Published:** 2022-08-11

**Authors:** Liangliang Meng, Bin Wu, Xiao Zhang, Xiaobo Zhang, Yingtian Wei, Xiaodong Xue, Zhongliang Zhang, Xin Zhang, Jing Li, Xiaofeng He, Li Ma, Yueyong Xiao

**Affiliations:** ^1^ Department of Radiology, the First Medical Center, Chinese PLA General Hospital, Beijing, China; ^2^ Department of Radiology, Chinese PAP Beijing Corps Hospital, Beijing, China; ^3^ Department of MRI, Affiliated Hospital, Logistics University of Chinese Peoples Armed Police Forces, Tianjin, China; ^4^ Department of Anesthesia and Surgery, the First Medical Center, Chinese PLA General Hospital, Beijing, China

**Keywords:** microwave ablation (MWA), lung cancer, anesthesia, pleural, pain

## Abstract

**Objectives:**

To explore the efficacy and safety of local pleural anesthesia (LPA) for relieving pain during microwave ablation (MWA) of pulmonary nodules in the subpleural regions.

**Materials and Methods:**

From June 2019 to December 2021, 88 patients with 97 subpleural nodules underwent percutaneous CT-guided MWA. Patients were divided into two groups according to whether LPA was applied; 53 patients with local pleural anesthesia during MWA; and 35 patients with MWA without LPA. The differences in technical success, pre-and post- and intra-operative visual analog scale (VAS) pain scores, complications of the procedure, and local progression-free survival (LPFS) between the two groups were assessed. Thus, to evaluate the efficacy and safety of MWA combined with LPA for treating subpleural nodules.

**Results:**

In this study, the procedures in all patients of both groups achieved technical success according to pre-operative planning. There was no statistically significant difference in the pre-operative VAS pain scores between the two groups. Intra-operative VAS scores were significantly higher in the non-LPA (NLPA) group than in the LPA group. They remained significantly higher in the NLPA group than in the LPA group during the short postoperative period. Analgesics were used more in the NLPA group than in the LPA group intra- and postoperatively, with a statistically significant difference, especially during the MWA procedures. The overall LPFS rates were 100%, 98.333%, 98.333%, and 98.333% at 1, 3, 6, and 12 months postoperatively in the LPA group and 100%, 97.297%, 94.595%, and 94.595% postoperatively in the NLPA group, respectively. Tumor recurrence occurred in one and two patients with lung adenocarcinoma in the LPA and NLPA groups. The incidence of pneumothorax was significantly higher in the NLPA group (25,714%, 9/35) than in the LPA group (15.094%, 8/53), and there were three cases of pleural effusion (blood collection) and one case of pulmonary hemorrhage in the NLPA group.

**Conclusion:**

Percutaneous CT-guided MWA is a safe and effective treatment for subpleural pulmonary nodules. Applying a combined LPA technique can reduce the patient’s pain and complications during and after the MWA. The long-term efficacy must be verified in more patients and a longer follow-up.

## 1 Introduction

Lung cancer is one of the most prevalent malignancies worldwide, with the highest morbidity and mortality in males in China (1, 2). For a long time, surgical resection was the preferred or even the only treatment for early-stage lung cancer (3). According to the latest clinical guideline, radical surgical resection is the preferred local treatment for stage I and II non-small cell lung cancer (NSCLC) (4). However, the most obvious limitation of surgery is the damage to the patient’s pulmonary function reserve, especially in older patients with poor underlying lung function (5). In addition, the surgery is more costly and the recovery time for patients is usually long. The popularity of thoracoscopy has reduced the damage to patients, but there are still some shortcomings (6). However, for patients who are inoperable or unwilling to have a surgical resection, CT-guided percutaneous thermal ablation plays an increasingly crucial role in treating lung tumors. It has comparable treatment efficacy to surgical procedures, but the overall damage to the patient is minimal, and the cost is significantly less than conventional surgery (7).

Recently, numerous studies confirming the effectiveness and safety of minimally invasive percutaneous ablations in the treatment of lung tumors (8). However, the efficacy of minimally invasive CT-guided treatment is closely related to the surgeon’s procedure plan and requires more skill and experience from the surgeon. Incomplete ablation of the tumor leads to recurrence or even recoil. Heavy pain during thermal ablation, which seriously affects the procedure, is the most reason for incomplete ablation and the most common postoperatively complication (9). The pleura is rich in nerves, and a slight irritation can lead to a severe pleural reaction. Therefore, the most serious difficulty in thermal ablation of sub-pleural nodules is achieving complete inactivation of the tumor safely and comfortably. Okuma reported that patients were more likely to undergo pain during radiofrequency ablation (RFA) when the distance between the tumor nodule and the thoracic wall was <1 cm (10). Pleuritic pain is the most common complication during thermal ablation (9). Some patients experience intolerable pain during local anesthetic ablation, which can usually be relieved by reducing the treatment power or shortening the ablation time. Still, this management may lead to incomplete ablation and increase the risk of tumor recurrence. Intravenous anesthesia can adversely affect pulmonary ventilation and increase the chance of infection (9). Microwave ablation (MWA) is more likely to cause pain in patients than RFA due to the difference in the underlying mechanism of thermogenesis (11).

The nerves that innervate the parietal pleura are the intercostal and phrenic nerves, which are somatosensory (12, 13). Stimulating the nerves on the parietal pleura can cause significant pain, radiating along the intercostal nerve to the chest and abdominal wall and the phrenic nerve to the neck and shoulder (13). The nerves in the visceral pleura come from the pulmonary plexus and enter the lung surface *via* the pulmonary hilum along the outer membrane of the pulmonary artery, peribronchial and lobular septa visceral sensory nerves with a high threshold for pain. Due to the proximity to the pleura, heat conduction will involve the pleura during radiofrequency ablation, which will cause pain in milder cases and partially cause a pleural reaction, leading to the failure of the procedure (13).

Our institution has performed many thermal ablations of pulmonary nodules in recent years, including MWAand radiofrequency ablation. It has accumulated considerable unique experience, including in the thermal ablation of pulmonary nodules in the adjacent pleural area. Our team has gradually worked out a method of local pleural anesthesia for the difficult treatment of subpleural pulmonary nodules, ensuring complete tumor ablation while minimizing the patient’s pain and damage to the pleura. This study aimed to evaluate the efficacy and safety of local pleural anesthesia in the MWA treatment of subpleural pulmonary nodules.

## 2 Materials and methods

### 2.1 Patients enrollment

This retrospective study was approved by the Ethics Committee of the Chinese PLA general hospital. Fifty-three patients with subpleural pulmonary nodules who underwent CT-guided percutaneous MWA with local pleural anesthesia in our hospital from June 1, 2019, to December 31, 2021, were retrospectively analyzed, and another 35 patients with subpleural pulmonary nodules who underwent MWA without local pleural anesthesia during this period were also recruited in this study as a control group. Because of the study’s retrospective nature, informed consent was waived by the Ethics Committee of our institution. This paper does not contain any person’s data in any form. We reviewed the hospital records and radiographic data of these patients.

Based on our prior knowledge, we defined subpleural nodules as pulmonary nodules at any distance within 1.0 cm from the pleura (10, 14). The specific inclusion criteria for patients were: (1) tumor margin within 1 cm from the pleura, including pleural apex, cribriform pleura, diaphragmatic pleura and mediastinal pleura. The distance of the lesion from the skin puncture point should be less than 15 cm to ensure that the antenna can reach the center of the lesion; (2) single pulmonary nodule less than 3.0 cm; (3) number of pulmonary malignancies three or less; (4) patients who refused or were not suitable for surgical resection. Exclusion criteria included severe lung infection, coagulation dysfunction, severe pulmonary failure, uncontrollable angina pectoris, cardiac arrhythmia, congestive heart failure, or a history of implantable cardiac devices. After screening, a total of 88 patients (51 male and 37 female, 42-90 years) were enrolled in the study. The baseline and tumor characteristics of the patients are summarized in **Table 1**. These 88 patients had 97 subpleural pulmonary nodules and were treated with MWA. Patients were divided into groups LPA and NLPA according to whether they applied local pleural anesthesia.

**Table 1:** Patient clinical information and demographics for all patients.

	Group LPA	Group NLPA	p-value
Age (years) (range)	66.04±10.427 (42-86)	67.00±10.350 (51-90)	0.672
Gender			0.449
Male	29	22	
Female	24	13	
Nodule size (mm) (range)	18.50±5.625 (9-30)	16.43±5.928 (6-30)	0.088
≤10	4	7	
10<d≤20	35	21	
20<d≤30	21	9	
Nidus location			0.288
Left upper lung lobe	14	10	
Left lower lung lobe	6	8	
Right upper lung lobe	20	9	
Right middle lung lobe	2	3	
Right lower lung lobe	18	7	
Body position			
Supine	18	13	
Left lateral	24	16	
Right lateral	18	8	
Type of lesion			0.017
Solid	45	19	
Sub-solid	15	18	
Pathology			0.210
Squamous cell carcinoma	4	1	
Adenocarcinoma	25	21	
Pulmonary metastases	22	7	
Unknown	6	7	
Other	3	1	
Preoperative VAS score	0.83±0.753 (0-3)	1.06±0.906 (0-3)	0.260
0-2	52	33	
3-5	1	2	
6-10	0	0	
Distance from tumor to pleura (mm)	2.92±2.999 (0-9)	4.68±2.819 (0-9)	0.004
0-4	45	18	
5-10	15	19	
Ablation parameters (range)			
Power (W)	30.75±6.163 (20-40)	28.784±6.056 (20-40)	0.164
Duration (min)	7.409±2.653 (3-16.5)	7.526±2.351 (4.33-14)	0.695

LPA, local pleural anesthesia, NLPA, non-local pleural anesthesia, VAS, visual analog scale.

The vast majority of nodules had definite pathology by percutaneous biopsy before the MWA procedures, while some patients’ pathology was obtained during the ablation. Only 13 nodules could not have definite pathology. For patients without definite pathology before surgery, we performed the MWA procedure at the patients’ strong request and with the ethics committee’s consent because the imaging signs suggested a high possibility of malignancy.

### 2.2 Local pleural anesthesia adjuvant MWA procedure

#### 2.2.1 MWA procedure

The procedures were performed with a spiral computed tomography (CT) Scanner (Philips Brilliance Big Bore 16-layer, Philips, USA) for pre-operative localization, pre-operative planning, intraoperative monitoring of ablation procedures, assessment of treatment response, and observation of complications. MWA of pulmonary nodules was performed using the “Tumor Microwave Ablation Therapy System” and the matched sterile disposable MWAantennas from Cannyon Medical Technology Co. The ablation antennas were selected according to the position, the size of the lesion, and the distance of the lesion from the skin. The procedures were usually performed by three experienced interventionalists with at least five years of experience in MWA treatment at our institution. There were also two permanent CT technicians and two surgical nurses who cooperated with the procedures. The patient’s position during the procedure was determined according to the tumor’s location. The patient’s position was fixed using shaped pads, pads, and baffles to ensure that the patient could remain comfortably in the same position for a long period.

Before the procedure, intravenous access was first established by the operating nurse to facilitate intraoperative emergency or therapeutic drug administration; the patients were kept on continuous low-flow oxygen (3-5L/min) through a nasal tube. The procedure monitored patients with cardiac monitoring, oxygen saturation, and heart rate. The patient’s blood pressure was measured every 5 minutes. All the above actions were taken to monitor the patient’s status during the procedure and provide timely intervention and treatment.

All patients included in this study underwent MWAunder local anesthesia. After local disinfection of the skin of the puncture point, 10-20ml of 1% lidocaine would be applied for local anesthesia. Local anesthesia before antenna entry acts primarily subcutaneously at the entry point and at the point where the microwave antenna would soon pass through the pleura. Lidocaine was usually mixed with 0.9% saline at a ratio of 1:1.

After local anesthesia, the ablation applicators should be placed in a predefined position using a stepwise method. The ablation parameters were selected according to the tumor size, location, morphology, adjacent structures, and the approach. The gauge size of the MWAapplicator was 17G, and the antenna length was 10cm or 15cm. Double-antenna clamping was usually chosen for the treatment when the tumor was larger than 2 cm, or the lesion was dense and difficult to penetrate. The power and duration of ablation vary according to the lesion’s density and size. Complete ablation refers to complete necrotic lesions of the local tumor tissue and a possible cure through thermal ablation. The complete ablation in the MWA procedure was usually indicated by the appearance of the halo sign around the lesion (15). When the ground-glass opacity around the lesion exceeded the nodule border by more than 5 mm, the tumor could be considered completely covered by the ablation area, complete ablation should be achieved, the procedure could be stopped, and the antennas should be removed. CT scan or enhanced imaging observed complete ablation of the tumor, and the ablated area appeared as a clear non-enhanced area (15). If the patient experienced significant pain during or after the procedure, analgesics should be given intravenously, usually 50 mg of flurbiprofen axetil injection.

#### 2.2.2 Application of local pleural anesthesia

To reduce the damage to the pleura after microwave ablation, we creatively applied the method of local pleural anesthesia. Select an appropriate syringe or percutaneous puncture to anesthetize the pleura adjacent to the lesion according to the positional relationship between the lesion and the pleura. Specifically, the site of administration of local pleural anesthesia should be in the pleura closest to the subpleural nodule. When the length of the anesthesia needle was less than 3 cm, a 5ml syringe and needle could be used directly for anesthesia, and when it was longer than 3 cm, a 20G or 21G puncture needle (PTC needle) should be used for anesthesia. The specific method was first to measure the distance from the skin of the puncture site to the target pleura on the CT image and to use a 20G or 21G puncture needle to lay the needle after anesthesia for the subcutaneous and puncture access, and to place the needle tip outside the pleura. All MWAprocedures in this study was done under local anesthesia, and patients were kept awake throughout the procedure. The local pleura is adequately anesthetized before the start of microwave ablation. If the patient felt any painful stimuli during the ablation procedure, regional lidocaine analgesia should be administered immediately until the patient achieved complete pain-free. During administration, local CT images should be used to confirm adequate infiltration of the pleura by the anesthetic. When the amount of lidocaine reached 20ml, ropivacaine was usually used to avoid the toxic side effects of the patient due to an overdose of anesthesia. In addition, lidocaine is a short-acting local anesthetic with faster dispersion by local injection, whereas ropivacaine has better analgesia and lasts longer than lidocaine, so ropivacaine was also given to some patients who were not satisfied with lidocaine analgesia.

#### 2.2.3 Pain assessment

All patients were evaluated before, during, and after the MWA procedure using a visual analog scale (VAS) system, which was applied in previous literature (16–18). The patients were scored and recorded on the VAS scale before, during, and 24 hours after the procedure to compare the differences in pain degrees between the two groups. Depending on the score, they were graded as mild (VAS 0-2 points), moderate (VAS 3-5 points), or severe (VAS 6-10 points). We obtained the above information through in-patient records and follow-up records. We compared the differences in pain levels between the two groups preoperatively, intraoperatively and postoperatively. In addition, since some patients used two antennas during ablation, we also compared the differences in pain levels between single- and double-antennas patients within each group separately to analyze the effect of the number of MWA antennas on pain levels.

### 2.3 Treatment efficacy

Local efficacy after MWAtreatment was assessed according to the criteria drafted by Ye et al. (15). Post-treatment follow-up imaging by CT was performed to evaluate the ablation outcome. Follow-up CT observed the efficacy of treatment and the presence of residual or recurrent lesions. Chest-enhanced CT was usually used to assess the outcome of the procedure. A complete lack of enhancement in the ablation zone is defined as technical success. Complete ablation of the tumor was observed by CT scan or enhanced imaging, and the ablated area appeared as a clear non-enhanced area. Incomplete ablation was observed as a nodular enhancement at the tumor margin or enlargement of the lesion in some areas around the tumor. Irregular nodal enhancement in the ablation zone was considered a recurrence or residual tumor. CT scan can also be used to observe the size and morphological changes of the tumor ablation area. Local progression-free survival (LPFS) was used to describe the absence of disease progression after treatment. The incidence of LPFS at different follow-up times was assessed according to the modified solid tumor response evaluation criteria. Due to the various tumors included in this study, the efficacy evaluation was only validated using LPFS. The time of LPFS was calculated from the day of the MWA procedure.

### 2.4 Complications

CT scan would be performed intra- and postoperatively to exclude complications. Common complications of MWA include pneumothorax, hemorrhage, and pleural effusion. Complications should be reported using the updated SIR classification criteria table so they can be consistently classified according to severity. Major complications result in severe morbidity and disability (e.g., resulting in unexpected organ loss), which increase the level of care or result in prolonged hospitalization (SIR C-E). This includes any condition requiring blood transfusion or interventional drainage. All other complications are considered to be minor.

### 2.5 Statistical analysis

Statistical analysis was performed using SPSS software version 26.0 (Chicago, IL, USA). Pearson chi-square and Fisher’s exact tests were used for categorical variables and Mann–Whitney U tests for continuous variables. A Paired two-sample T-test was used to compare the differences in VAS scores before and after MWA in each group separately. Two independent samples t-tests were used to compare differences in clinical information and outcomes related to patients in the two groups (LPA vs. NLPA). If the data did not conform to a normal distribution, then a non-parametric test was used. The statistical threshold was set at a *P*-value of less than 0.05.

## 3 Results

### 3.1 Patient clinical information and demographics

Eighty-eight patients with 97 subpleural pulmonary lesions were included in this study, and all patients underwent CT-guided microwave ablation. There were 53 patients in the LPA group and 35 in the NLPA group. The mean patient age was 66.04 ± 10.43 in the LPA group and 67.00 ± 10.27 mm in the NLPA group. The mean tumor size was 18.50 ± 5.63 mm in the LPA group and 16.43 ± 5.93 mm in the NLPA group. Both groups had the highest proportion of pathological types of lung adenocarcinoma (Group LPA: 24/53, Group NLPA: 21/35), followed by lung metastases. Thirty-one patients were placed in the supine position, and 66 were placed in the lateral position during the MWA procedure.

The detailed clinical information of all patients is shown in **Table 1**.

### 3.2 Technical success rate

**Figure 1:**
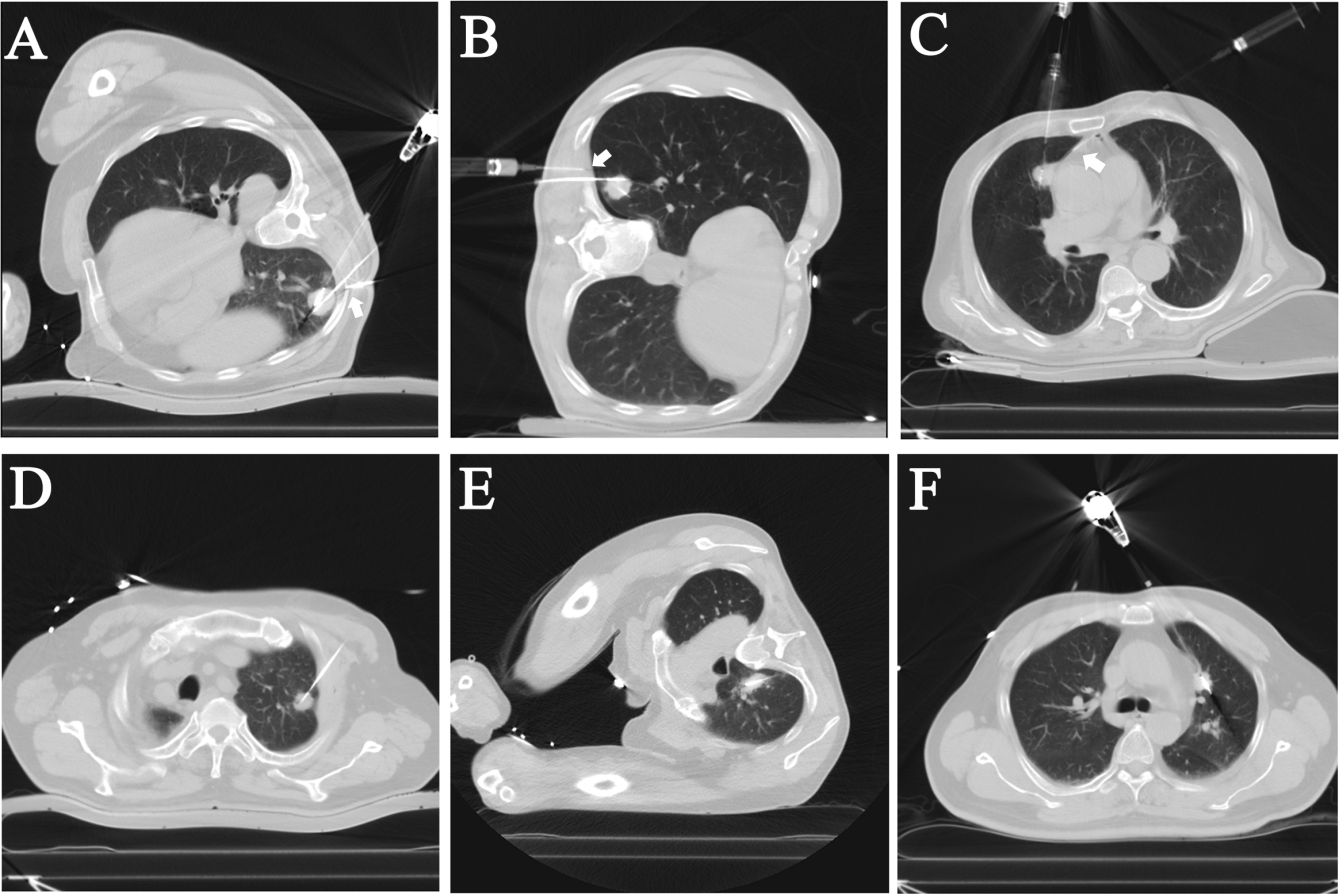
Intraoperative CT images of patients who underwent local pleural anesthesia (LPA) or not during the microwave ablation (MWA) procedures. **(A–C)** In the LPA group, the needle and syringe used for local pleural anesthesia can be seen on the CT images. The tips of the anesthesia needles (Arrows) were visualized on the CT as reaching the subpleural and used to administer the anesthesia. **(D–F)** The NLPA group’s ablation antennas were located in the center of the nodules without local pleural anesthesia.

This study performed CT-guided MWA on 97 subpleural pulmonary nodules in 88 patients. All 88 patients were treated according to the protocol, and ablation was performed by single- or double-antenna, and intra- and post-operative enhanced CT assessment of the ablated tumor area showed complete coverage of the tumor without significant enhancement of the tumor area, suggesting the technical success of MWA. In the LPA group, local pleural anesthesia was applied, and the anesthetic needle reached the designated position and worked in all patients (**Figures 1A–C**). In the NLPA group, no local pleural anesthesia was used during MWAin all patients (**Figures 1D–F**). The technical success rate of the LPA group was 100%; the technical success rate of the NLPA group was also 100%. (**Table 2**)

**Table 2:** Technique efficacy and follow-up of LPFS.

Group	Characteristics	Patients	Technical success	Technique efficacy (LPFS)		
				≥3 Months	≥6 Months	≥12 Months
Group LPA n=60	Pathology					
	Adenocarcinoma	25	25	24	24	24
	Squamous cell carcinoma	4	4	4	4	4
	Pulmonary metastases	22	22	22	22	22
	Nodule size (mm)					
	0<d≤10	4	4	4	4	4
	10<d≤20	35	35	34	34	34
	20<d≤30	21	21	21	21	21
	Distance to pleura (mm)					
	0-4	45	45	44	44	44
	5-10	15	15	15	15	15
Group NLPA n=37	Pathology					
	Adenocarcinoma	22	22	21	20	20
	Squamous cell carcinoma	1	1	1	1	1
	Pulmonary metastases	7	7	7	7	7
	Nodule size (mm)					
	0<d≤10	7	7	7	7	7
	10<d≤20	21	21	21	20	20
	20<d≤30	9	9	8	8	8
	Distance to pleura (mm)					
	0-4	18	18	17	16	16
	5-10	19	19	19	19	19

LPFS, Local Progress Free Survival; LPA, local pleural anesthesia; NLPA, non-local pleural anesthesia.

### 3.3 Treatment efficacy

**Figure 2:**
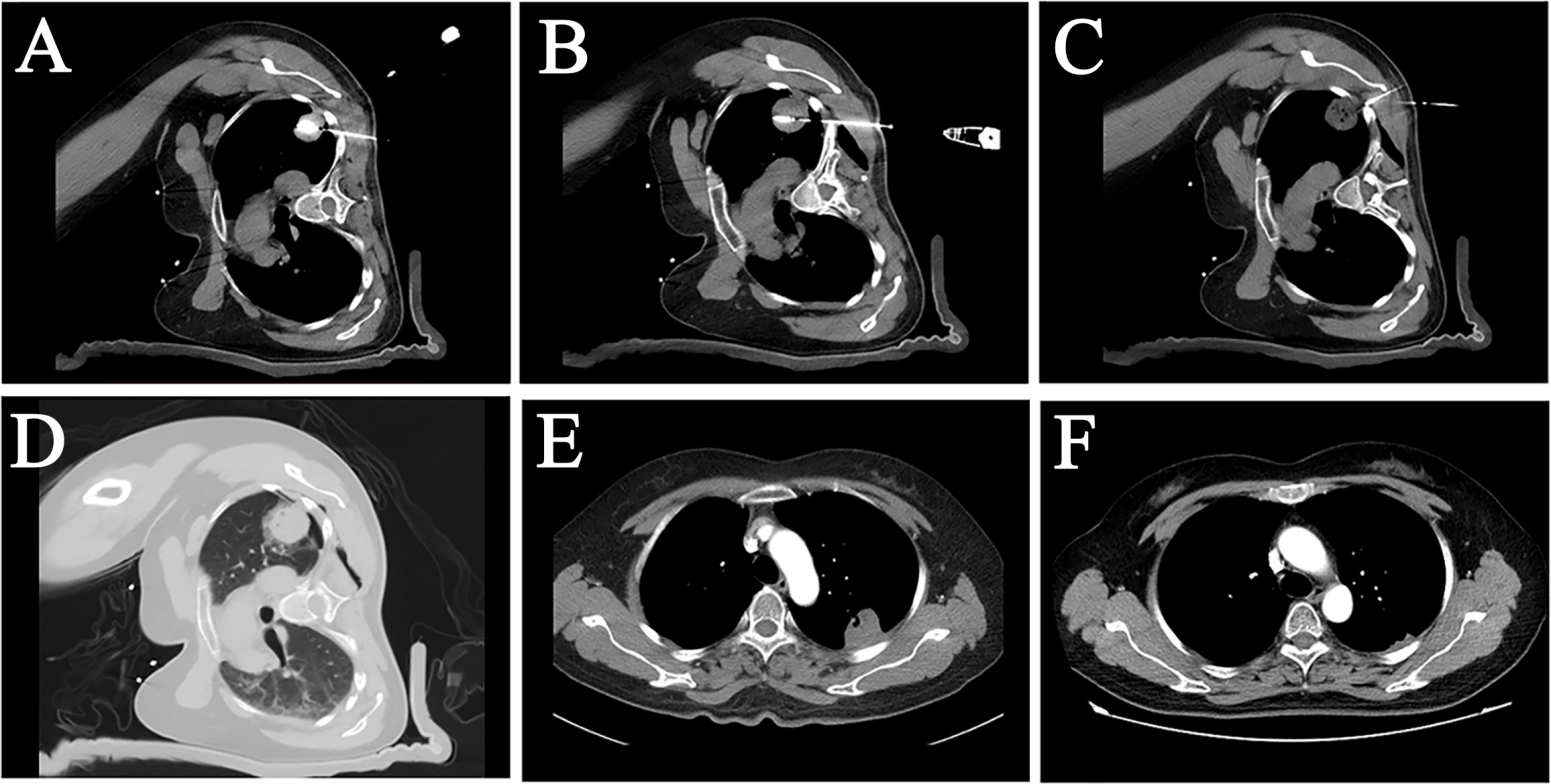
A female patient, 50 years old, with pulmonary metastasis from osteosarcoma, which was eradicated with an MWA procedure with LPA. **(A, B)** CT image showed a sizeable subpleural metastasis in the upper lobe of the patient’s left lung. Two MWA antennas were used on the patient at different layers to perform MWAof this metastatic tumor. **(C)** Local pleural anesthesia was used on the patient during the treatment. The tip of the anesthesia needle was placed in the subpleural area near the tumor. **(D)** An immediate postoperative CT scan showed a “halo sign” surrounded by a ground-glass density around the lesion. **(E)** A follow-up examination three months after surgery showed that the lesion was smaller than before, and no abnormal enhancement was seen inside the lesion. **(F)** Twelve months later, the tumor largely disappeared, suggesting that the pulmonary metastasis had achieved radical ablation.

**Figure 3:**
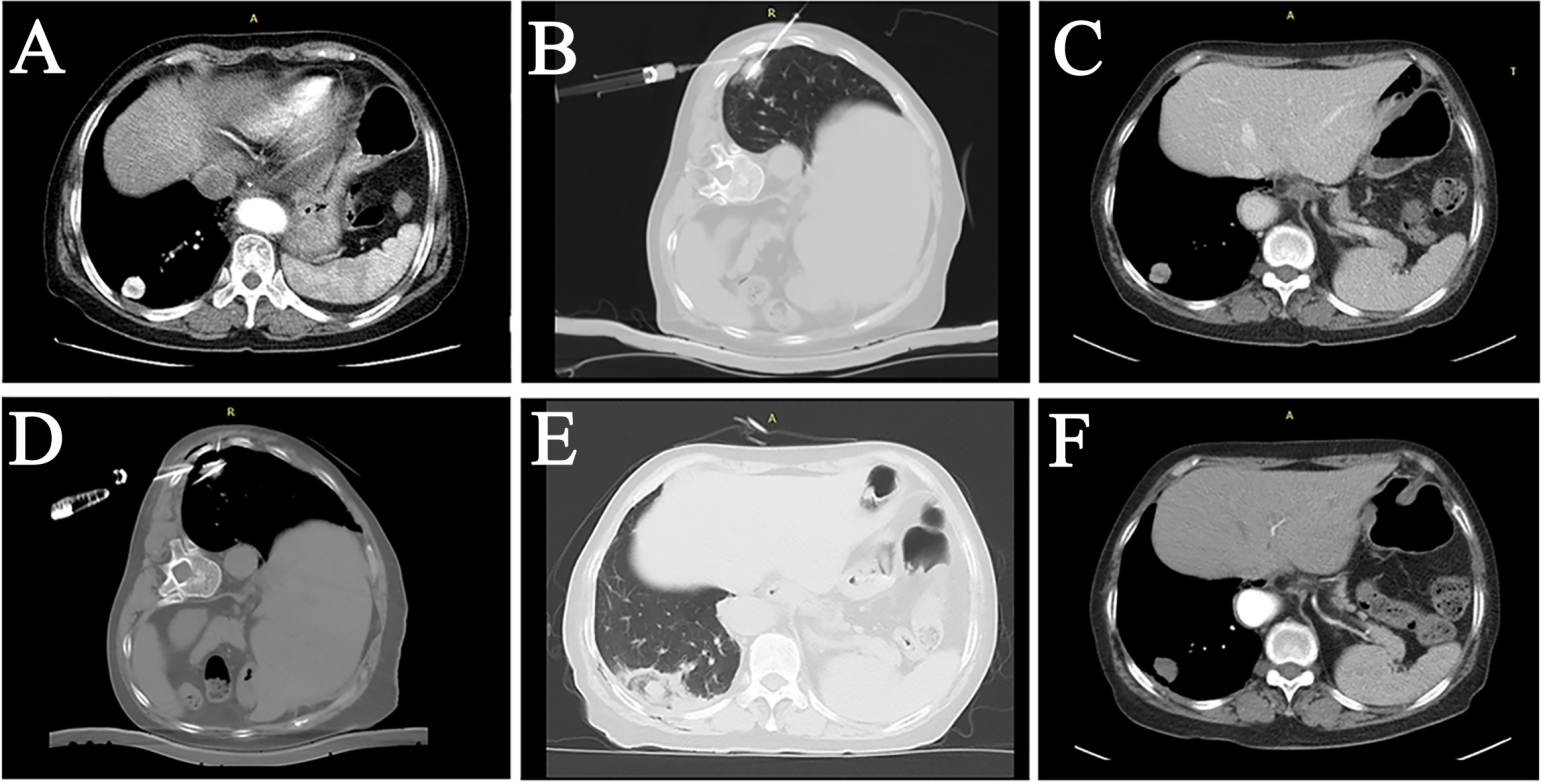
A female patient, 72 years old, with lung adenocarcinoma in the right lower lung lobe, underwent secondary ablation after the first incomplete ablation. **(A)** The tumor was located in the subpleural area with significant enhancement. **(B)** First MWA treatment combined with LPA. **(C)** Follow-up CT three months after the intervention showed significant inhomogeneous enhancement within the tumor. **(D)** Re-ablation of the tumor was performed using two MWA antennas combined with LPA. **(E)** Postoperative CT showed a “halo sign” surrounding the tumor. **(F)** 3-month follow-up after the secondary ablation indicated complete tumor ablation.

We observed the efficacy of the treatment through post-operative CT follow-up. The postoperative outcomes were followed up at one day, one month, three months, six months, and 12 months after the MWA procedure. In both groups, most patients experienced complete ablation of the lesions after microwave ablation, which gradually shrank or even disappeared over time (**Figure 2**). Only one patient in the LPA group showed abnormal internal enhancement three months after treatment, which was considered an incomplete ablation, and a second MWA operation was performed electively (**Figure 3**). In the NLPA group, two lesions showed inhomogeneous enhancement 3 and 6 months after the procedure, and incomplete tumor ablation and recurrence were considered. Each of these patients underwent secondary ablation.

The technical outcomes and short-term follow-up of the two groups are shown in **Table 2**, in which the overall LPFS rates were 100%, 98.333%, 98.333%, and 98.333% at 1, 3, 6, and 12 months postoperatively in the LPA group and 100%, 97.297%, 94.595%, and 94.595% postoperatively in the NLPA group, respectively (**Table 2**).

### 3.4 Pain degree assessment

We applied only a small amount of ropivacaine (less than 10 ml) after 20 ml of lidocaine in only three patients in the study, and the postoperative VAS score was performed 24 h after the MWA, so our anesthetist considered that this would not have an impact on the accuracy of the VAS score.

Since the data of each group did not satisfy a normal distribution, the Mann-Whitney U test was used to determine whether there was a difference in VAS scores between the two groups at pre-, intra-, and post-operative periods. The shape of the VAS score distribution was not consistent between the two groups as judged by the histogram. The mean rank order of VAS in the pre-operative LPA group was 42.17, and the mean rank order of VAS in the NLPA group was 48.03. The Mann-Whitney U test showed that the difference in VAS between the pre-operative LPA and NLPA groups was not statistically significant (U=804.000, *P*=0.260). The mean rank order of VAS was 35.12 in the LPA group and 58.7 in the NLPA group, and the Mann-Whitney U test showed that there was a statistically significant difference between the VAS of the LPA (2.36 ± 1.039) and NLPA (3.86 ± 1.574) groups during the procedure (U=430.500, *P*<0.001). The mean rank order of VAS in the post-operative LPA group was 40.04, and the mean rank order of VAS in the NLPA group was 51.26. The Mann-Whitney U test showed a statistically significant difference between the VAS in the post-operative LPA and NLPA groups (U=691.000, *P*<0.031) (**Table 3**).

**Table 3:** Differences of VAS scores between the two groups.

Period	Group	Number	Mean	Std. Deviation	Median	Mann-Whitney U	*P*-value
Pre-operation	LPA	53	0.83	0.753	1	804.000	**0.260**
	NLPA	35	1.06	0.906	1		
Intra-operation	LPA	53	2.36	1.039	2	430.500	**0.000**
	NLPA	35	3.86	1.574	4		
Post-operation	LPA	53	1.21	0.906	1	691.000	**0.031**
	NLPA	35	1.51	0.818	2		

LPA, local pleural anesthesia; NLPA, non-local pleural anesthesia; VAS, visual analog scale.

The Mann-Whitney U test was also used to determine whether there were differences in VAS scores in each group at the time points, respectively. The analysis showed statistically significant differences in VAS between the LPA group preoperatively, intraoperatively, and postoperatively (*P*<0.05). The intraoperative VAS was the highest, while the postoperative score was slightly higher than the pre-operative score (*P*=0.027). Similar to the results of the LPA group, there were statistically significant differences in VAS between the two groups in the NLPA group preoperatively, intraoperatively, and postoperatively (*P*<0.05) (**Supplementary Table 1**).

In addition, some patients used analgesics during and after the MWA procedure, and statistics revealed that the use frequency of intraoperative analgesics in the NLPA group was significantly higher than that in the LPA group (*P*=0.036) (**Table 4**).

**Table 4:** Efficacy and complications of microwave ablation in the two groups.

	Group LPA(n=53)	Group NLPA(n=35)	*P*-value
Days of hospitalization	6.00±1.373	6.60±1.594	0.51
Technical success			
Yes	53	35	
No	0	0	
Technique Efficacy			
Yes	52	33	
No	1	2	
Intra-operative analgesics			0.036
Yes	8	12	
No	45	23	
Post-operative analgesics			0.082
Yes	5	8	
No	48	27	
Post-procedure VAS score (range)	1.21±0.906 (0-4)	1.51±0.818 (0-3)	0.031
Follow-up duration (months) (range)	12.19±3.157	13.43±3.509	0.104
Recurrences			
Yes	1	2	
No	52	33	
Complications			
Pleurodynia	0	1	
Pneumothorax	8	9	
Pleural effusion	2	3	
Pulmonary hemorrhage	1	1	
Pneumonia	0	0	
Others	0	0	

LPA, local pleural anesthesia; NLPA, non-local pleural anesthesia; VAS, visual analog scale.

We also analyzed the effect of the number of antennas on the pain degree. Given that the data did not conform to a normal distribution, we used the Mann-Whitney U test to conduct the analyses in the LPA and NLPA groups separately. The Mann-Whitney U test showed no statistically significant difference in pain levels between single and dual antenna patients in the LPA group intraoperatively (U=310, *P*=0.511). The NLPA group showed the same results, with no statistically significant difference in pain levels between single and dual antenna patients intraoperatively (U=142.5, *P*=0.756). No statistically significant differences were also found between both groups in the postoperative analysis (*P*>0.05) (**Supplementary Table 2**).

### 3.5 Adverse events/complications

The SIR scoring system was used to assess the complications of MWA surgery (17). All 88 patients had no severe complications. In the LPA group, pneumothorax occurred in 8 patients (15.094%, 8/53), 4 of whom were evaluated for large pneumothorax volume and underwent tube drainage; 2 patients had Pleural effusion, and one patient underwent bleeding around the lesion, and the related complications were effectively controlled after the application of hemostatic drugs and other medical treatments (**Supplementary Figure 1**).

In the NLPA group, nine patients had a pneumothorax (25.714%, 9/35), 6 of whom were evaluated and underwent tube drainage due to the large volume of pneumothorax; 3 patients had different amounts of pleural effusion, and the related complications were effectively controlled after the application of hemostatic drugs and other medical treatments. The relevant results are detailed in **Table 4**.

## 4 Discussion

The subpleural pulmonary nodule itself was not precisely defined. Okuma et al. reported that when the distance between the tumor and the chest wall was less than 1 cm, patients may feel severe pain from heat transfer from the target area to the pleura during thermal ablation therapy (10). Local pleural anesthesia can protect adjacent structures and reduce pain without affecting the patient’s respiratory function. This study defined nodules less than 1 cm from the pleura as subpleural nodules, and subpleural nodules were ablated using MWA. To explore the safety and efficacy of local pleural anesthesia in the MWA of subpleural nodules, we grouped patients with subpleural nodules treated by MWA for nearly two years into group LPA and group NLPA. In this study, 88 patients with 97 subpleural nodules were treated with MWA, and local pleural anesthesia was used to address the pain during the ablation process. The use of local pleural anesthesia significantly reduced intraoperative and postoperative pain compared to a small number of patients who did not have local pleural anesthesia. All patients tolerated the entire procedure better. In addition, we routinely applied a mixture of lidocaine and saline to increase the diffusion thickness and extent of local pleural anesthesia within the range of safe anesthetic doses and provided better protection to the pleura. The results showed that local pleural anesthesia significantly reduced the intraoperative pain of patients, and the patients’ post-operative pain got to be alleviated accordingly. In addition, the use of intra- and post-operative intravenous analgesics was also less in the LPA group than in the NLPA group, suggesting that LPA played a promising analgesic role in the intra- and post-operative periods. Painkillers such as flurbiprofen have substantial side effects such as neurotoxicity and are contraindicated in patients with digestive tract ulcers or bleeding (19).

We are the first to report the use of local pleural anesthesia in MWA. The local pleural anesthesia mentioned here differs from the anesthesia for puncture procedures such as biopsy. Because of the short biopsy time and the absence of distance pain caused by thermal stimulation, the local anesthesia done during the biopsy is sometimes acceptable for less than complete pleural anesthesia. However, the pain caused by thermal ablation is very intense, and the analgesic effect is not good enough during microwave ablation treatment. The present study is thorough and continuous anesthesia of the local pleura adjacent to the lesion during ablation using local anesthetics lidocaine or ropivacaine, which is an innovation in pain management of microwave ablation and has been reported very rarely in the literature before. Most of the related literature has focused on the use of artificial pneumothorax and has not explicitly reported the use of local pleural anesthesia. Artificial pneumothorax was considered an effective pain relief and tissue protection method during thermal ablation and tumor biopsy (9, 20, 21). Previously, artificial pneumothorax was used in RFA of pulmonary adjacent mediastinal lesions, and effective therapeutic results and effective protection of proximal mediastinal structures could be achieved using artificial pneumothorax (9). Yang et al. compared MWA in 17 patients with and 20 without artificial pneumothorax and reported that artificial pneumothorax significantly reduced pain during and after the procedure (17). However, in some cases, including patients with pleural adhesions and severe respiratory insufficiency, artificial pneumothorax may not be effective or may not be tolerated by the patient. In addition, excessive compression of the lung parenchyma can alter the RF’s electrical conductivity and thermal conductivity, affecting the ablation efficiency and leading to increased lung tissue damage (22). In particular, the excessive collapse of lung tissue can lead to subpleural lesions closer to the pleura, and ultimately thermal ablation may lead to complications such as pleural fistula. In addition, the application of artificial pneumothorax requires a high level of technical skill, and improper operations can lead to severe complications such as respiratory distress and subcutaneous emphysema; in addition, for some specific lesions, the adjustment of position and gas pushing are not effective in forming an ideal gas isolation zone. An excessive amount of artificial pneumothorax may also lead to lung collapse, increase the difficulty of antenna transfer, produce more damage to lung tissue during treatment, and even lead to severe complications such as pleural fistula. In addition, a small amount of gas does not eliminate the pain caused by MWA due to the preferable conductivity of the gas to heat. The application of artificial pneumothorax in the treatment of subpleural lung cancer has a considerable limitation.

Pneumothorax is the most common complication of RFAand MWA (23, 24). Rika Yoshimatsu et al. suggested that delayed and recurrent pneumothorax occurs more likely when the GGO around the lesion adheres to the pleura (24). A small amount of pneumothorax can usually resolve independently, while a larger amount may require chest tube drainage. In this study, the incidence of pneumothorax was significantly lower in the LPA group than in the NLPA group. We believe this may be due to the better compliance and tolerance of local pleural anesthesia patients, who are less prone to violent coughing and excessive respiratory dynamics that may lead to pneumothorax. In addition, very few patients in both groups had thoracic bleeding and a small amount of intrapulmonary hemorrhage. Local pleural anesthesia also did not increase the chance of intercostal artery injury or bleeding, indicating the safety of local pleural anesthesia.

MWA is an effective minimally invasive treatment for eradicating pulmonary nodules (25, 26) and has achieved better outcomes in treating subpleural nodules (14, 17). Previously, for subpleural lesions, ablation was usually performed at lower power for a longer period to protect the pleura and reduce pain as much as possible. This may result in incomplete tumor ablation for larger lesions and affect the ablation outcome. The higher power usually leads to pain and the inability to tolerate the procedure. The duration of MWA depends mainly on the changes in lung tissue density around the lesion of ablation (27). Usually, we use the halo sign of ground-glass density around the lesion and the range of ground-glass density beyond the edge of the lesion by more than 5 mm as the sign of complete ablation. Although there was no significant difference in the duration of ablation between the two groups in this study, the mean treatment power was somewhat higher in the LPA group than in the NLPA group, suggesting that patients who underwent local pleural anesthesia were more tolerant of the higher MWA power to achieve complete ablation. This study again demonstrated the efficacy of MWAin primary and metastatic subpleural pulmonary nodules. Only 3 of all 88 patients had a postoperative recurrence, and all were eradicated after repeated MWA. Due to the different pathology of subpleural nodules, only LPFS was used in this study to observe the efficacy of local lesion ablation. The LPFS rate was slightly higher in the LPA group than in the NLPA group, suggesting that LPA combined with MWA may produce better efficacy. And this needs to be validated with more patients and longer follow-ups in the future.

There are some limitations of this study. First, the degree of pain was assessed using the VAS score, a highly subjective score system that may affect the accuracy of the results. Second, this study was a retrospective analysis. More confounding factors such as the application of ropivacaine in a few patients in this study brought bias to the results; Second, because of the different pathologies of pulmonary nodules, we only observed the short-term efficacy of MWA by comparing the LPFS of the patients. The long-term efficacy of MWA needs to be validated further; Third, Due to the limitation of patients, the control group was not selected using the more scientific propensity score matching method, and the relevant results need to be further validated in a larger sample in the future.

## 5 Conclusion

In conclusion, CT-guided MWA is a safe and effective minimally invasive treatment for subpleural pulmonary nodules. Local pleural anesthesia can significantly reduce patients’ intra- and postoperative pain during MWA, facilitate the implementation of the surgical plan, complete ablation of the lesion, and reduce the occurrence of complications such as pneumothorax. The long-term efficacy must be verified in more patients and a longer follow-up.

## Data availability statement

The raw data supporting the conclusions of this article will be made available by the authors, without undue reservation.

## Ethics statement

The studies involving human participants were reviewed and approved by Ethics Committee of the Chinese PLA general hospital. Written informed consent for participation was not required for this study in accordance with the national legislation and the institutional requirements.

## Author contributions

LM, WB and YYX conceived and designed the study. LM and BW drafted the entire manuscript. XZ and XBZ are responsible for patient information acquisition and post-operative follow-up. XDX and XFH are responsible for imaging and image processing. YTW and XNZ reviewed and revised the manuscript. LMA and ZLZ participated in figures and table preparation. JL and YYX guided the article revision and the selection of statistical methods. All authors contributed to the manuscript revision and approved the submitted version.

## Conflict of interest

The authors declare that the research was conducted in the absence of any commercial or financial relationships that could be construed as a potential conflict of interest.

## Publisher’s note

All claims expressed in this article are solely those of the authors and do not necessarily represent those of their affiliated organizations, or those of the publisher, the editors and the reviewers. Any product that may be evaluated in this article, or claim that may be made by its manufacturer, is not guaranteed or endorsed by the publisher.

## Supplementary material

Supplementary Figure 1 Complications of MWA for subpleural pulmonary nodules. **(A)** After ablation of the tumor in the right lung, a CT scan revealed a large pneumothorax with decreased oxygen saturation in the patient. **(B)** The patient was placed on a chest tube for drainage. **(C)** A slight intraoperative intrathoracic bleeding was found in the patient’s right lower lobe lung lesion. **(D)** After treatment was completed, the scan after antenna removal revealed an increase in pleural blood accumulation compared to the previous one, and symptomatic therapies such as hemostatic drugs were given, and the patient was closely monitored.

Supplementary Table 1 Differences of VAS in the peri-operative periods in each group.

Supplementary Table 2 Differences in VAS between single and dual antennas in each group.
