# Supplementary figures and images for: Microwave ablation with local pleural anesthesia for subpleural pulmonary nodules: our experience
Source: Front Oncol. 2022 Aug 11;12:957138. doi: 10.3389/fonc.2022.957138 (PMC9411023; doi:10.3389/fonc.2022.957138)

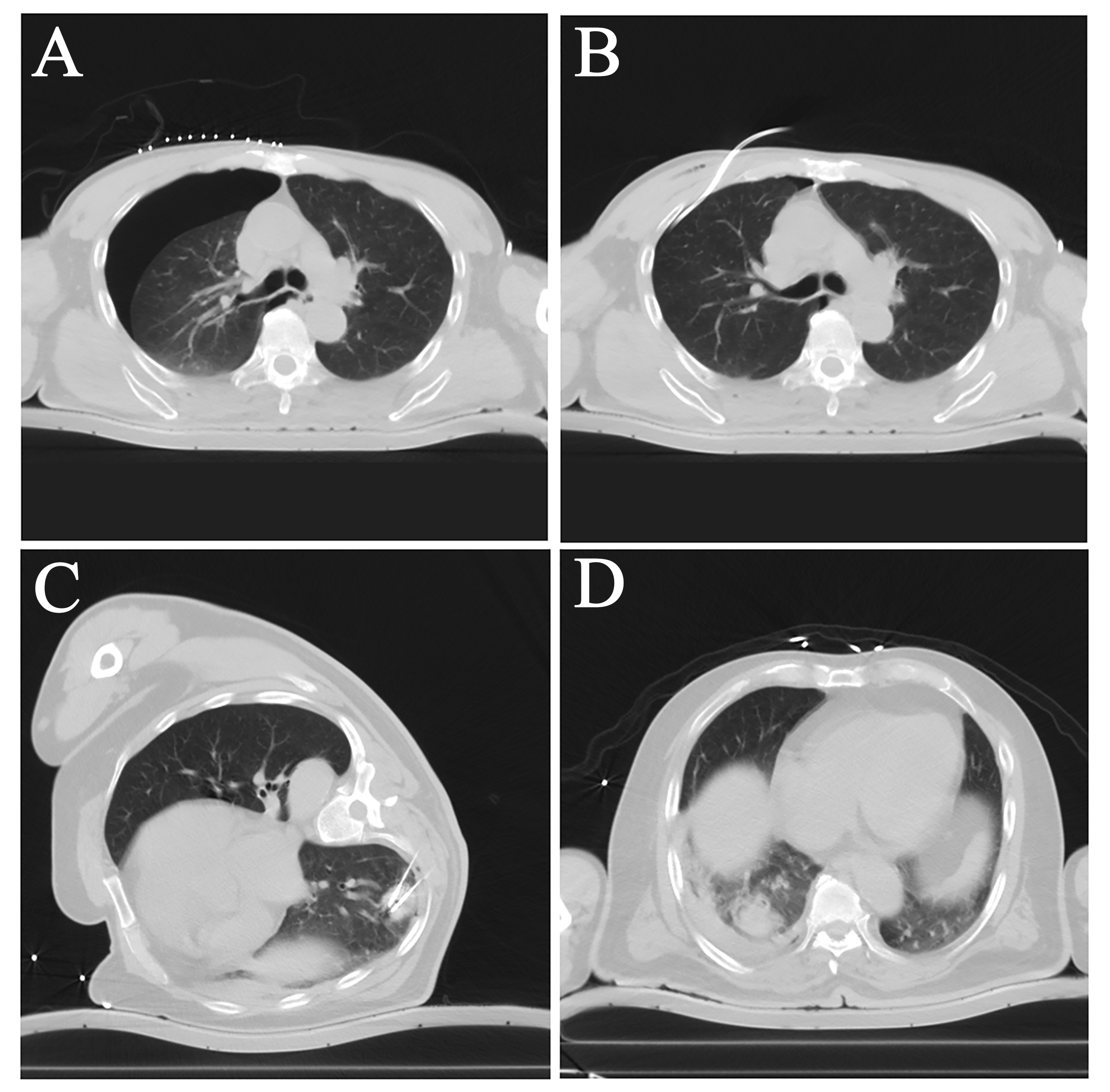

Supplement: Supplementary Figure 1 — Complications of MWA for subpleural pulmonary nodules. (A) After ablation of the tumor in the right lung, a CT scan revealed a large pneumothorax with decreased oxygen saturation in the patient. (B) The patient was placed on a chest tube for drainage. (C) A slight intraoperative intrathoracic bleeding was found in the patient’s right lower lobe lung lesion. (D) After treatment was completed, the scan after antenna removal revealed an increase in pleural blood accumulation compared to the previous one, and symptomatic therapies such as hemostatic drugs were given, and the patient was closely monitored. [file Image_1.tif]
