# Supplementary material for: Microwave ablation with local pleural anesthesia for subpleural pulmonary nodules: our experience
Source: Front Oncol. 2022 Aug 11;12:957138. doi: 10.3389/fonc.2022.957138 (PMC9411023; doi:10.3389/fonc.2022.957138)
Supplement: Supplementary Table 1 — Differences of VAS in the peri-operative periods in each group. [file Table_1.docx]

Table S1 Differences of VAS in the peri-operative periods in each group

| Group |  | Number of cases | Mean VAS | Std. Deviation | | Mean Rank | Mann-Whitney U | *P*-Value |
| --- | --- | --- | --- | --- | --- | --- | --- | --- |
| Group LPA | Pre-operation | 53 | 0.83 | | 0.753 | 33.79 | 360.000 | 0.000 |
|  | Intra-operation | 53 | 2.36 | | 1.039 | 73.21 |  |  |
|  | Intra-operation | 53 | 2.36 | | 1.039 | 69.12 | 576.500 | 0.000 |
|  | Post-operation | 53 | 1.21 | | 0.906 | 37.88 |  |  |
|  | Pre-operation | 53 | 0.83 | | 0.753 | 47.4 | 1081.000 | 0.027 |
|  | Post-operation | 53 | 1.21 | | 0.906 | 59.6 |  |  |
| Group NLPA | Pre-operation | 35 | 1.06 | | 0.906 | 20.19 | 76.500 | 0.000 |
|  | Intra-operation | 35 | 3.86 | | 1.574 | 50.81 |  |  |
|  | Intra-operation | 35 | 3.86 | | 1.574 | 49.44 | 124.500 | 0.000 |
|  | Post-operation | 35 | 1.51 | | 0.818 | 21.56 |  |  |
|  | Pre-operation | 35 | 1.06 | | 0.906 | 30.3 | 430.500 | 0.024 |
|  | Post-operation | 35 | 1.51 | | 0.818 | 40.7 |  |  |

Notes: LPA, local pleural anesthesia, NLPA, non-local pleural anesthesia, VAS, visual analog scale.
