# Supplementary material for: Microwave ablation with local pleural anesthesia for subpleural pulmonary nodules: our experience
Source: Front Oncol. 2022 Aug 11;12:957138. doi: 10.3389/fonc.2022.957138 (PMC9411023; doi:10.3389/fonc.2022.957138)
Supplement: Supplementary Table 2 — Differences in VAS between single and dual antennas in each group. [file Table_2.docx]

Table S2 Differences of VAS between single and dual antennas in each group

| Group |  |  | Number of cases | Mean VAS | Std. Deviation | | Mean Rank | Mann-Whitney U | *P*-Value |
| --- | --- | --- | --- | --- | --- | --- | --- | --- | --- |
| Group LPA | Intra-operation | Single antenna | 30 | 2.37 | | 0.850 | 28.17 | 310.000 | 0.511 |
|  |  | Dual antennas | 23 | 2.26 | | 1.214 | 25.48 |  |  |
|  | Post-operation | Single antenna | 30 | 1.27 | | 1.015 | 26.80 | 339.000 | 0.907 |
|  |  | Dual antennas | 23 | 1.26 | | 1.010 | 27.26 |  |  |
| Group NLPA | Intra-operation | Single antenna | 19 | 3.95 | | 1.545 | 18.50 | 142.500 | 0.756 |
|  |  | Dual antennas | 16 | 3.75 | | 1.653 | 17.41 |  |  |
|  | Post-operation | Single antenna | 19 | 1.79 | | 1.134 | 18.58 | 141.000 | 0.731 |
|  |  | Dual antennas | 16 | 1.56 | | 1.031 | 17.31 |  |  |

Notes: LPA, local pleural anesthesia, NLPA, non-local pleural anesthesia, VAS, visual analog scale.
